# Interface and material engineering for zigzag slab lasers

**DOI:** 10.1038/s41598-017-16968-0

**Published:** 2017-12-01

**Authors:** Fei Liu, Siyu Dong, Jinlong Zhang, Hongfei Jiao, Bin Ma, Zhanshan Wang, Xinbin Cheng

**Affiliations:** 1 MOE Key Laboratory of Advanced Micro-Structured Materials, Shanghai, 200092 China; 2 Institute of Precision Optical Engineering, School of Physics Science and Engineering, Tongji University, Shanghai, 200092 China; 3 IFSA Collaborative Innovation Center, Shanghai Jiao Tong University, Shanghai, 200240 China

## Abstract

Laser damage of zigzag slab lasers occurs at interface between laser crystal and SiO_2_ film. Although an additional HfO_2_ layer could be used to manipulate electric-field on the crystal-film interface, their high absorption and polycrystalline structure were unacceptable. SiO_2_ was then doped in HfO_2_ to suppress its crystallization and to achieve low absorption by annealing. Hf_x_Si_1−x_O_2_ nanocomposite layers were then inserted between laser crystal and SiO_2_ film to minimize electric-field at crystal-film interface. Laser damage resistance of this new architecture is two times higher than that of traditional zigzag slab lasers.

## Introduction

The zigzag slab architecture is widely used in high power lasers. It can restrain thermally induced lensing and birefringence to obtain high output energy and exceptional beam quality^1–3^. Extremely high laser power has been obtained using zigzag slab laser architecture by Northrop Grumman^4,5^. Usually, the total internal reflection (TIR) in zigzag slab architecture is achieved by depositing lower index coatings on the surfaces of a laser crystal. Moreover, the coating is connected to the heat sink to take waste heat away^6^. When laser passes a zigzag path between two TIR surfaces in the crystal, strong electric-field intensity (EFI) is created. The coating must have low absorption, otherwise the joint effect of EFI and absorption will induce strong thermal effects and decrease the beam quality. SiO_2_ is the dominant material for zigzag slab lasers due to its extremely low absorption from near ultraviolet to near infrared region.

When the operating fluence of laser is higher and higher, the laser-induced damage becomes a severe issue. The crystal-film interface is the most vulnerable to laser damage, because massive nano-sized absorbers are created in vicinity of crystal-film interface due to surface contamination, polishing residues in the subsurface of the laser crystal, extraction of defects during coating deposition, or microstructure mismatch between the crystal and the film^7–10^. Laser damage at crystal-film interface is induced by the joint contribution of nano-sized absorbers and strong EFI^11–15^. The laser-induced damage threshold (LIDT) can be increased either by removing the massive nano-sized absorbers near crystal-film interface or by reducing EFI in vicinity of the crystal-film interface. Because it is quite challenging to remove all these nano-sized absorbers at crystal-film interface, the approach that reduces EFI is very promising. However, until now, the laser crystal with a SiO_2_ coating is the only used configuration. No other slab laser architecture has been proposed to minimize EFI at crystal-film interface to improve LIDT. It is highly desirable to explore novel slab laser architecture to replace the traditional one.

Our previous studies have investigated the high reflection (HR) coatings that were irradiated from crystal-film interface^11,16^. It was found that adding a high index coating between the laser crystal and SiO_2_ thin film could minimize the EFI at crystal-film interface and increase the laser damage resistance. However, there is a crucial difference between HR coatings and TIR coatings. Standing-wave EFI is created in HR coatings, whereas, evanescent-wave EFI develops in the SiO_2_ layer for the TIR case. What a role that evanescent-wave EFI plays in laser damage and the experimental studies of interfacial damage of TIR surfaces have been rarely reported. In this work, the laser damage characteristics of traditional zigzag slab lasers was first investigated. Then, interface and material engineering were performed to find novel architecture of the zigzag slab lasers with increased LIDT.

## Results and Discussion

### Laser damage characteristics of the traditional zigzag slab lasers

**Figure 1:**
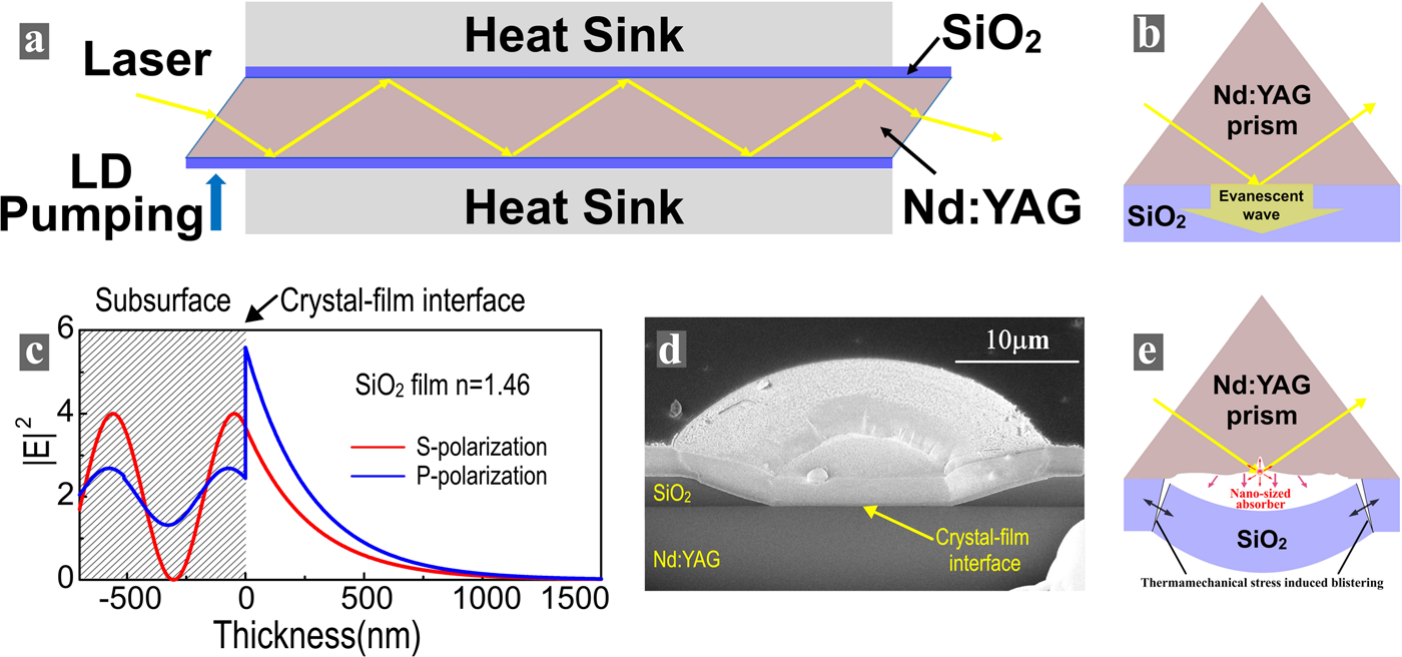
(**a**) Diagram of a zigzag slab laser. (**b**) Schematic, (**c**) EFI distributions, (**d**) damage morphology and (**e**) a phenomenological damage model of a Nd:YAG prism coated with a SiO_2_ layer.

The traditional configuration of the zigzag slab Nd:YAG laser working at 1064 nm is given in Fig. 1(a). The dimensions of slab are about 1.7 mm high, 67 mm long, 11 mm wide and 45° angle cut at the end. The crystalline orientation of Nd:YAG crystal is (111). The doping concentration of Nd:YAG crystal is about 0.05%. A 3 μm SiO_2_ coating is deposited on the Nd:YAG crystal to achieve TIR and it is also connected to heat sink. The working laser passes a zigzag path between two TIR surfaces in the crystal and the pumping laser enters from the end of the crystal. It is difficult to directly study the laser damage characteristics of the zigzag slab lasers, therefore, an analog using Nd:YAG prisms coated with SiO_2_ film was studied instead, as shown in Fig. 1(b). The Nd:YAG prisms with 0.05% doping and a size of 30 millimeters to a side were used for this study. The incident angle at crystal-film interface is set as 55° to achieve TIR.

The EFI distribution within the Nd:YAG crystal and SiO_2_ thin film is given in Fig. 1(c). The standing-wave EFI is created in the subsurface of the crystal and the evanescent-wave EFI evolves in the SiO_2_ coating. Because S-polarized EFI rather than P-polarized EFI could be reduced to zero at abrupt interfaces, only S-polarized laser damage is discussed here. The difference between S-polarized and P-polarized laser damage can be found in our previous work^16^.

The LIDT testing was performed using 1064 nm, 10 ns pulses from a Nd:YAG laser having a TEM_00_ mode. The raster scan method was used to obtain the LIDTs^17,18^. The LIDT of the traditional TIR configuration is about 10 ± 2 J/cm^2^ (Table 1). The cross-sectional view of one representative damage morphology was revealed using a focus ion beam (FIB) technique, as shown in Fig. 1(d). It can be seen that the depth of the damage site is equal to the thickness of the SiO_2_ coating, meaning that the laser damage initiated from the crystal-film interface. Both standing-wave EFI and evanescent-wave EFI are close to their maximum value at the crystal-film interface where nano-sized absorbers are concentrated, laser damage occurred preferentially at this interface, which reflects that evanescent-wave EFI at least plays a similar role in laser damage like standing-wave EFI. The formation of the damaged crater can be explained phenomenologically, as given in Fig. 1(e). The nano-sized absorbers at crystal-film interface is heated by the laser pulse to a very high temperature at which the defect-surrounding matrix is converted to the absorbing medium through photoionization by ultra violet (UV) radiation^19^, thermionic emission of electrons^20^ and the heat-transfer-induced band-gap collapse^21^. When the materials are heated to the melting temperature, thermomechanical damage occurs and results in blistering of the coating from Nd:YAG crystal.

**Table 1:** LIDTs of three TIR configurations.

TIR configurations	Traditional TIR	TIR using HfO_2_ layer	TIR using Hf_x_Si_1−x_O_2_ layer
LIDT (J/cm^2^, 1064 nm, 10 ns)	10 ± 2	6 ± 2	18 ± 2

### Minimizing EFI at crystal-film interface using HfO_2_ high index material

**Figure 2:**
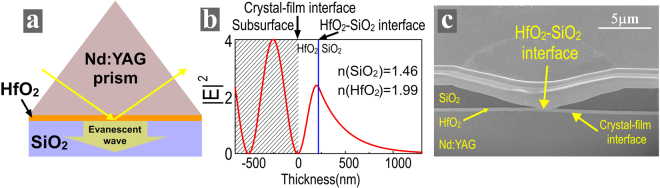
(**a**) Schematic, (**b**) EFI distribution and (**c**) damage morphology of a Nd:YAG prism coated with HfO_2_ and SiO_2_ films.

EFI in the traditional TIR configuration is fixed, additional approach must be found to engineer EFI at the crystal-film interface to improve LIDT. The HfO_2_ film was explored to engineer the EFI at the crystal-film interface due to its optimal properties in the region from near ultraviolet to near infrared^22^. It was inserted between the Nd:YAG crystal and SiO_2_ coating to minimize EFI at crystal-film interface, as shown in Fig. 2(a). By choosing thickness of the HfO_2_ layer to be 230 nm, a destructive interference was created at the crystal-film interface and the EFI at this interface was decreased to zero, as given in Fig. 2(b). The side effects of adding a HfO_2_ layer is that evanescent-wave EFI at the new HfO_2_-SiO_2_ interface is quite strong.

SiO_2_ and HfO_2_ coating on large optics is usually prepared using electron beam evaporation (EBE) process. However, the packing density of coatings prepared by EBE process is also low, and a lot of voids exist in the coatings, making their environmental stability and thermal conductivity poor. Here, the SiO_2_ and HfO_2_ coating was prepared using Ion assisted deposition (IAD) to achieve dense microstructure with good environmental stability and thermal conductivity. The absorption of SiO_2_ coatings prepared by IAD process could be very low, however, HfO_2_ coatings prepared by IAD process are always highly absorbing^23^. Its absorptivity was 2 orders higher than that of the SiO_2_ coating, as given in Table 2.

**Table 2:** Absorptivity of SiO_2_ film and HfO_2_ film annealed at different temperatures.

Films	SiO_2_ unannealed	HfO_2_ unannealed	HfO_2_ annealed at 400 deg	HfO_2_ annealed at 500 deg	HfO_2_ annealed at 600 deg
Absorptivity (cm^−1^)	3.7 × 10^−2^	3.1	7.0 × 10^−1^	5.2 × 10^−2^	4.5 × 10^−2^

The damage testing indicated that LIDT of this TIR configuration was only 6 ± 2 J/cm^2^ (Table 1) and it is even worse than that of the traditional TIR configuration. The cross-sectional view of the representative damage morphology was examined to explain the LIDT result. Figure 2(c) shows that the laser damage initiated from the HfO_2_-SiO_2_ interface. The reason is that the highly absorbing HfO_2_ coating leads to a concentration of intensive nano-sized absorbers at HfO_2_-SiO_2_ interface. Laser damage was created at this interface due to strong standing-wave EFI and evanescent-wave EFI here. The LIDT result reflects that the HfO_2_-SiO_2_ interface is even more vulnerable to the laser damage than the crystal-film interface.

**Figure 3:**
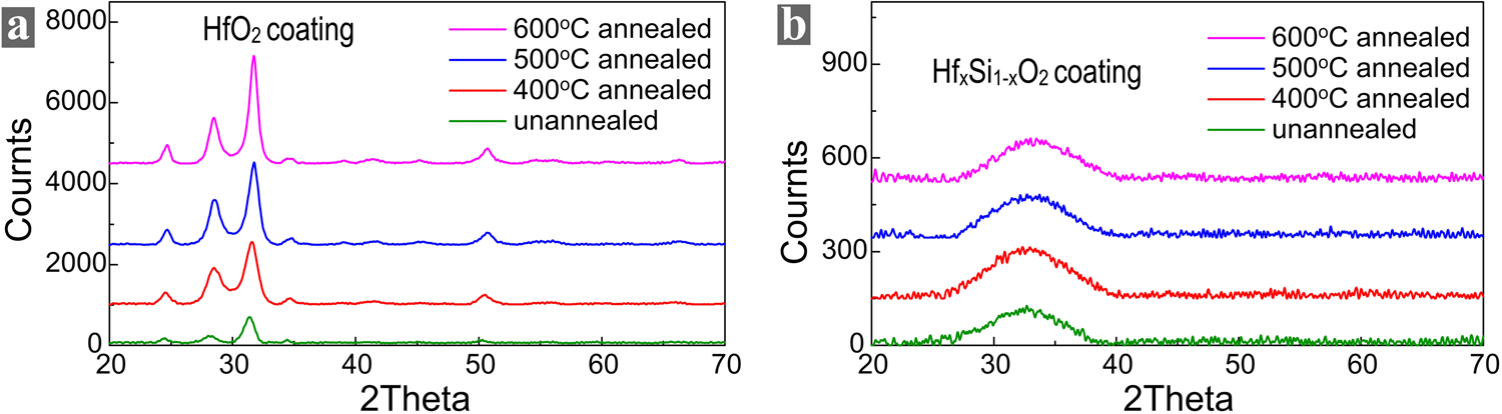
XRD curves of **(a)** HfO_2_ and **(b)** Hf_x_Si_1−x_O_2_ films annealed at different temperatures.

Our previous study showed that, when the absorption of both coatings is very low, the HfO_2_-SiO_2_ interface is much more resistant to laser damage than the crystal-film interface^11^. So, annealing was attempted to reduce the absorption of the HfO_2_ coating. The annealing temperature increases from 400 to 600 degrees centigrade with an increment step of 100 degrees centigrade. Table 2 shows that annealing at 600 degrees centigrade reduced the absorptivity of HfO_2_ coatings by two orders and its absorption reaches to the level that is close to that of SiO_2_ film. However, the cracking of the annealed HfO_2_ film was identified using a Normarski microscope, as shown in Supplementary Figure S1. Previous studies reported that the crystalline states of HfO_2_ films were changed during annealing, leading to volume change of the films, inducing strong associate stress and resulting in film cracking^22,24^. X-ray diffraction spectrometry (XRD) measurement revealed the change of the crystalline states during annealing, as given in Fig. 3(a). The XRD spectra shows that the HfO_2_ films are polycrystalline and these films most contain the monoclinic phase of HfO_2_ with preferred (111) orientation. The intensities of all the peaks become significantly higher when the annealing temperature increases. The large increase in peak intensity after high temperature annealing indicates that crystallite size has increased significantly. This leads to volume change of the films, induces strong associate stress and results in film cracking. As a result, annealing is not suitable to reduce the absorption of HfO_2_ films.

### Reducing EFI at crystal-film interface using Hf_x_Si_1−x_O_2_ nanocomposite

Two approaches have been proposed to suppress the crystallization of HfO_2_ coatings during annealing. Nanolaminate is one approach that adds thin amorphous layers periodically into HfO_2_ coatings to interrupt the crystallization^25^. However, this approach introduces many interfaces that will reduce the thermal conductivity and also reduce LIDT. It is not suitable to the current application. Nanocomposite is another approach that dopes amorphous material such as SiO_2_ in HfO_2_ to suppress crystallization^26–28^. It is possible to anneal Hf_x_Si_1−x_O_2_ nanocomposite to reduce its absorption without changing its microstructure.

**Figure 4:**
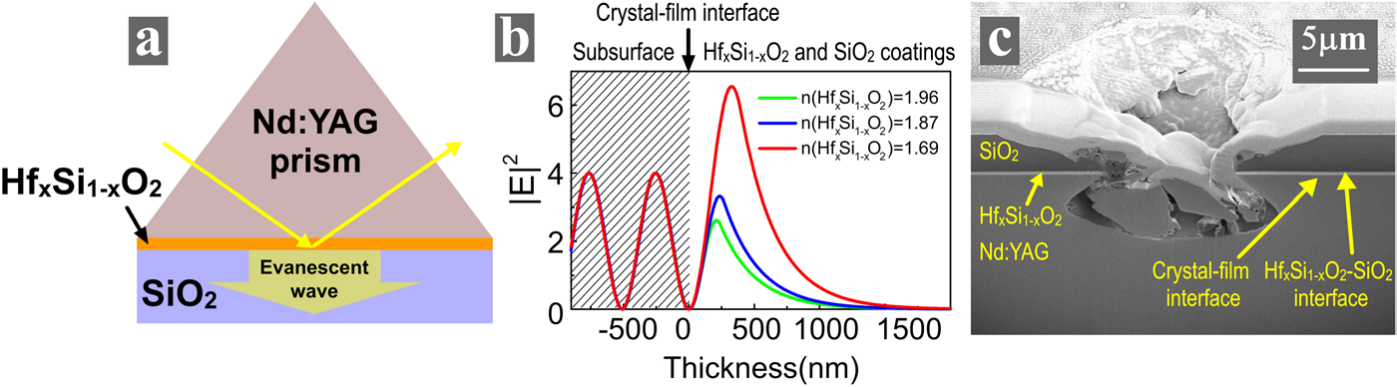
**(a)** Schematic, **(b)** EFI distributions and **(c)** damage morphology of a Nd:YAG prism coated with Hf_x_Si_1−x_O_2_ and SiO_2_ films.

Here, Hf_x_Si_1−x_O_2_ nanocomposite was prepared using a co-evaporation process with IAD. It was explored to engineer EFI at the crystal-film interface. The schematic of the configuration using Hf_x_Si_1−x_O_2_ nanocomposite is given in Fig. 4(a). The refractive indices of the Hf_x_Si_1−x_O_2_ nanocomposites can be varied in a wide range. Figure 4(b) presents the EFI distributions using Hf_x_Si_1−x_O_2_ nanocomposites with three different refractive indices. Although zero EFI at crystal-film interface could be achieved by selecting proper thickness of each Hf_x_Si_1−x_O_2_ nanocomposite layer, higher refractive index of the Hf_x_Si_1−x_O_2_ nanocomposite results in lower EFI in the coatings. It is beneficial to use the highest refractive index of the Hf_x_Si_1−x_O_2_ nanocomposite layer that does not crystalize at high annealing temperature. But the limitation is that the higher refractive index, the lower concentration of SiO_2_ in Hf_x_Si_1−x_O_2_ nanocomposite, and the worse resistant to crystallization at high annealing temperature. In our experiments, the refractive index of 1.87 at the wavelength of 1064 nm is the highest one that was still amorphous when the Hf_x_Si_1−x_O_2_ nanocomposite layer was annealed at 600 degrees centigrade. When its layer thickness is 280 nm, the EFI at the crystal-film interface is zero.

The unannealed Hf_x_Si_1−x_O_2_ nanocomposite layer was still highly absorbing. High temperature annealing effectively reduced its absorptivity to the level that is comparable to the SiO_2_ layer, as given in Table 3. The XRD measurement of the Hf_x_Si_1−x_O_2_ nanocomposite film annealed at different temperatures is given in Fig. 3(b). There are no sharp diffraction peaks in the XRD spectra of unannealed and annealed Hf_x_Si_1−x_O_2_ films, which shows that the Hf_x_Si_1−x_O_2_ films maintained amorphous microstructure during annealing.

**Table 3:** Absorptivity of Hf_x_Si_1−x_O_2_ film annealed at different temperatures.

Hf_x_Si_1−x_O_2_ Film	Unannealed	Annealed at 400 deg	Annealed at 500 deg	Annealed at 600 deg
Absorptivity (cm^−1^)	2.3	5.4 × 10^−1^	4.5 × 10^−2^	4.1 × 10^−2^

The LIDT of this novel TIR configuration is 18 ± 2 J/cm^2^ (Table 1), which is almost 2 times higher than that of the traditional TIR configuration (10 ± 2 J/cm^2^). A cross-sectional view of the representative damage morphology of this TIR configuration is given in Fig. 4(c). The damage site has a larger and more irregular shape, and its depth is much deeper than the thickness of coatings. It can be seen that the laser damage initiated from a deep location within the subsurface of Nd:YAG crystal. EFI at the crystal-film interface is zero, laser damage would not start from here. Moreover, the absorption of the annealed Hf_x_Si_1−x_O_2_ nanocomposite is very low, so the Hf_x_Si_1−x_O_2_-SiO_2_ interface has less nano-sized absorbers and is more resistant to laser damage compared to the crystal-film interface and the subsurface of the Nd:YAG crystal. It also reflects that the evanescent-wave EFI does not play a more detrimental role in laser damage than standing-wave EFI. As a result, only the nano-sized absorbers in the subsurface of the Nd:YAG crystal can trigger the laser-induced damage at the depth where peaks of EFI are located^11^. The absorptivity and density of nano-sized absorbers in the subsurface is much less than that at crystal-film interface, so the LIDT of this novel TIR configuration is almost 2 times higher than that of traditional one.

## Conclusion

Interface and material engineering were performed to improve LIDT of zigzag slab lasers. A layer of high index material of HfO_2_ was inserted between the Nd:YAG crystal and SiO_2_ film to reduce the EFI at the crystal-film interface to be zero. However, the absorption of HfO_2_ layer is too big and it was not practicable to reduce its absorption by annealing. Hf_x_Si_1−x_O_2_ nanocomposite was then synthesized using co-evaporation process to solve this issue. Hf_x_Si_1−x_O_2_ nanocomposite layer maintained its amorphous microstructure during annealing and achieved extremely low absorption. A novel configuration of zigzag slab lasers was proposed using Hf_x_Si_1−x_O_2_ nanocomposite layer, whose LIDT was about two times higher than that of the traditional configuration of zigzag slab lasers.

## Methods

### Sample preparation

The Nd:YAG prisms were first carefully cleaned by ultrasonic cleaning process. The SiO_2_ films and HfO_2_ films were prepared using ion assisted deposition (IAD) process, and the Hf_x_Si_1−x_O_2_ nanocomposite films were prepared using co-evaporation process with IAD techniques. A schematic of co-evaporation process with IAD techniques is shown in Supplementary Figure S2. The Hf_x_Si_1−x_O_2_ nanocomposite film was deposited on the substrates by co-evaporating HfO_2_ and SiO_2_ with IAD techniques. The two quartz crystal monitors were used to monitor the evaporation rates of two materials, and the two programmable masks were used to control the evaporation rates of two materials. The refractive indices of the Hf_x_Si_1−x_O_2_ nanocomposites can be varied in a wide range with different relative evaporation rates of two materials. The details of the cleaning and deposition processes can be found in our previous papers^18,29,30^. Annealing processes of the films were performed in air at atmospheric pressure at 400, 500 and 600 °C for 3 hours.

### Characterization methods

The LIDT testing was performed using 1.064 μm, 10 ns pulses from a Nd:YAG laser having a TEM 00 mode, a beam diameter of 1 mm and a repetition rate of 10 Hz. The raster scan method^17^ was used to obtain the LIDTs. The laser fluence increased to the “damage fluence” with a 2 J/cm^2^ increment. The damage morphologies may change during the raster scan test, so a single shot method was used to obtain representative damage morphologies. The damage morphologies were observed and characterized by an optical microscope, a scanning electron microscope (SEM) and a focus ion beam (FIB) equipment.

X-ray diffraction spectrometry (XRD) was used to characterize the crystallization of films with Cu K*α* as the X-ray source. Data were collected in the *θ*–2*θ* (locked couple) mode from 2*θ* of 20–70° with a step size of 0.02°. Optical microscope was used to identify the cracking of the annealed HfO_2_ coatings.

Photo-thermal technique was used to measure the absorptions of the films. The details of the absorption measurement can be found in our previous paper^23^. The absorptivity of coatings can be calculated by knowing the electric-field intensity (EFI) within them using the formula

1 $$A=\alpha \frac{{n}_{1}}{{n}_{0}}{\int }_{0}^{l}\frac{{E}^{2}(z)}{{E}_{0}^{2}}dz$$

where *A* is the absorption, *α* is the absorption coefficient, *n*_0_ and *n*_1_ are the refractive index of incident medium and film, *E* is the EFI and *l* is the thickness of the layer. The absorptivity of SiO_2_ film and HfO_2_ film prepared using electron beam evaporation (EBE) process and IAD process respectively is given in Supplementary Table S1.

## Electronic supplementary material

Supplementary information
