# Supplementary material for: Interface and material engineering for zigzag slab lasers
Source: Sci Rep. 2017 Dec 1;7:16699. doi: 10.1038/s41598-017-16968-0 (PMC5711792; doi:10.1038/s41598-017-16968-0)
Supplement: Supplementary file 1 — Supplementary information [file 41598_2017_16968_MOESM1_ESM.doc]

**Supplementary information for**

**Interface and material engineering for zigzag slab lasers**

**Fei Liu**^1,2,+^**, Siyu Dong**^1,2,+^**, Jinlong Zhang**^1,2,3^**, Hongfei Jiao**^1,2^**, Bin Ma**^1,2^**, Zhanshan Wang**^1,2,3^ **and Xinbin Cheng**^1,2,3,*^

^1^MOE Key Laboratory of Advanced Micro-Structured Materials, Shanghai, 200092, China

^2^Institute of Precision Optical Engineering, School of Physics Science and Engineering, Tongji University, Shanghai, 200092, China

^3^IFSA Collaborative Innovation Center, Shanghai Jiao Tong University, Shanghai 200240, China

*corresponding. chengxb@tongji.edu.cn


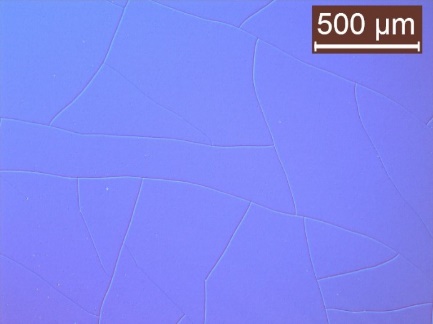

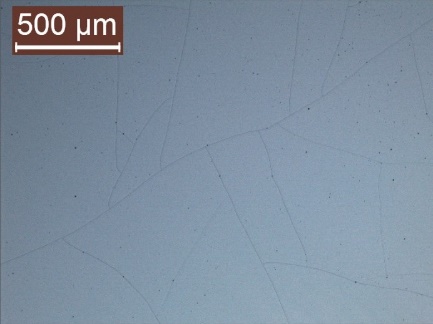

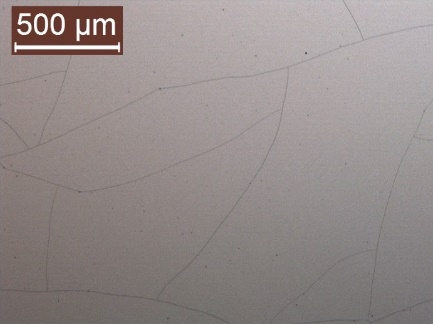


**Supplementary Figure S1. Optical micrograph of a cracked HfO_2_ film after 600****℃ annealing.** The HfO_2_ film is seriously cracked after 600℃ annealing.


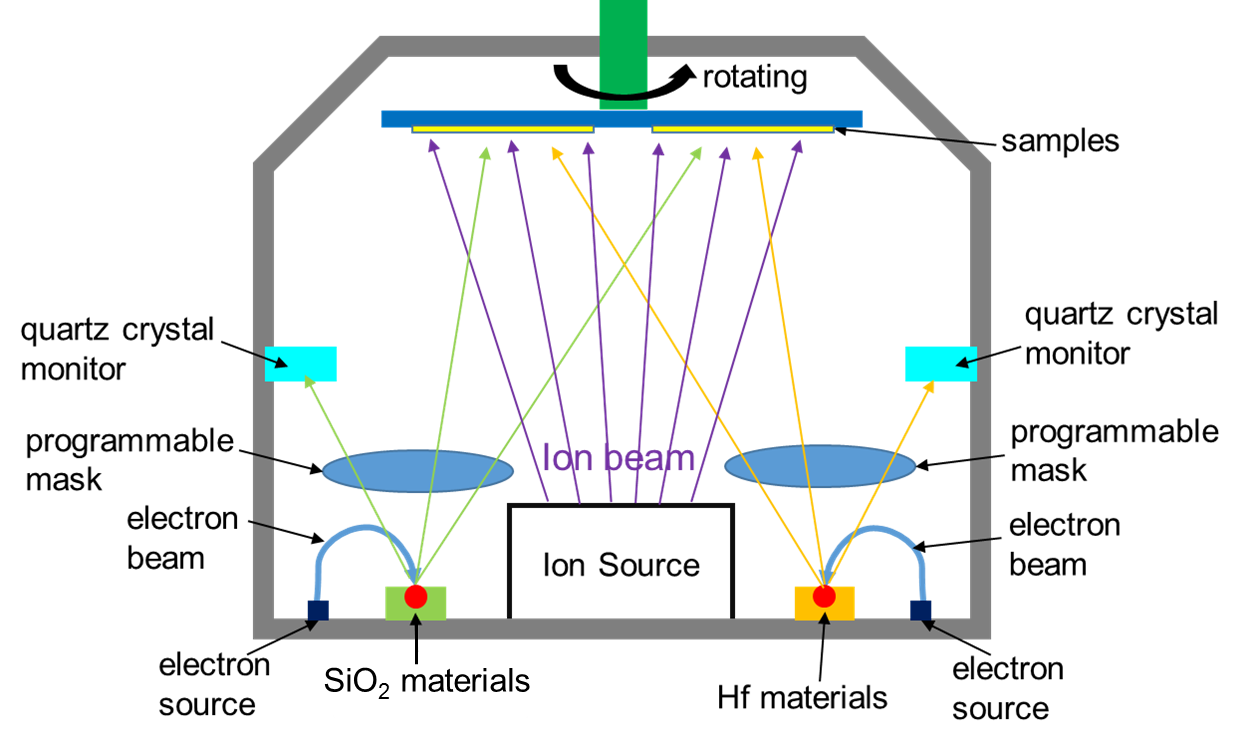


**Supplementary Figure S2. Schematic of co-evaporation process with IAD technique.**

| **film** | SiO_2_  (EBE process) | SiO_2_  (IAD process) | HfO_2_  (EBE process) | HfO_2_  (IAD process) |
| --- | --- | --- | --- | --- |
| **Absorptivity (cm^-1^)** | 3.4 x 10^-2^ | 3.7 x 10^-2^ | 6.3 x10^-2^ | 3.1 |

**Supplementary Table S1. Absorptivity of SiO_2_ and HfO_2_ films prepared using EBE and IAD process.** The absorption of SiO_2_ film prepared by IAD process is similar with that by EBE process. However, HfO_2_ film prepared by IAD process has much higher absorption than that by EBE process.
